# The Effectiveness of Pelvic Floor Muscle Exercise in Urinary Incontinence: A Systematic Literature Review and Meta-Analysis

**DOI:** 10.7759/cureus.45011

**Published:** 2023-09-11

**Authors:** Nicole S Parra, Arturo P Jaramillo, Jhon Zambrano, Diego Segovia, Javier Castells, Jhoanny C Revilla

**Affiliations:** 1 Internal Medicine, Pontificia Universidad Católica del Ecuador, Quito, ECU; 2 General Practice, Universidad Estatal de Guayaquil, Machala, ECU; 3 Emergency, Universidad Tech, Santa Cruz de Tenerife, ESP; 4 Medicine, Universidad Católica de Santiago de Guayaquil, Guayaquil, ECU; 5 Medicine, Universidad del Zulia, Maracaibo, VEN

**Keywords:** pelvic floor muscle exercises, male urinary incontinence, stress urinary incontinence, pelvic floor muscle training, urinary incontinence

## Abstract

Urinary incontinence (UI) is a prevalent health concern in females over 60, but it is prevalent in a smaller percentage of males. The medical and social elements of UI are crucial. This disorder may cause embarrassment and low self-esteem, reducing social and physical activities. Consequently, this may impair healthy aging. Researchers have shown that pelvic floor muscle training (PFMT) may improve UI symptoms in middle-aged, older, and young people. Clinical practice highlights the importance of PFMT for UI due to its low cost-effectiveness. To assess PFMT's overall efficacy, we conducted a systematic review of the literature (SRL) and a meta-analysis (MA) of randomized clinical trials (RCTs). The previous 10 years of published material were combed using the online databases the Cochrane Library, PubMed, and Google Scholar.

Eligible total studies were RCTs referring to the effectiveness of PFMT. The pooled incidence, risk ratio, and 95% confidence interval (CI) of the effectiveness of PFMT were calculated using the fixed effects model. Given the possibility of a between-study variance, we used the fixed effects model rather than the random effects model.

## Introduction and background

Urinary incontinence (UI) is a common condition experienced by a significant number of individuals globally, particularly adults. UI is used to talk about a condition of unintentional urine loss or leakage. The illness can affect individuals of both genders, although it is more commonly observed in females. There are various types of UI, including mixed incontinence, stress incontinence, and urge incontinence [1,2]. According to the definitions provided by the International Continence Society and the International Urogynecological Association, UI is described as the occurrence of urine leakage accompanied by a massive, hard-to-control, and strong urge to urinate. On the other hand, stress incontinence refers to the occurrence of urine leakage when sneezing, engaging in physical activity, or coughing [3]. Mixed incontinence, which refers to the combination of symptoms from two categories, is a common occurrence [4]. According to research, stress-related UI is a common condition that impacts a significant majority of individuals experiencing UI [5].

In the general female population, the common risk factors for UI include obesity, vaginal delivery, pregnancy, pelvic surgery, and age [2]. There is limited research available on the risk factors for UI in young females who have not given birth [5]. According to a study, there is a suggestion that having a low body mass index (BMI), having eating disorders, and engaging in minimal training could potentially contribute to UI [6]. UI can lead to restrictions in daily activities, sexual activity, and interpersonal relationships [7]. The negative impact on one's quality of life is evident when considering the emotional challenges that arise, including feelings of low self-worth, depression, unhappiness, and shame [8,9]. Many females who experience UI choose not to seek medical help due to the misconception that it is a common result of childbirth and getting older, rather than recognizing it as a significant health concern [10].

A comprehensive analysis of studies on pelvic floor muscle training (PFMT) has examined various non-surgical treatments for female UI, such as medication. Medication, behavioral therapies, biofeedback, and surgery are commonly used to treat UI. Behavioral therapy is often the preferred treatment for numerous patients experiencing stress incontinence. PFMT is the first therapeutic option in various cases due to its non-invasive nature and lack of associated risks. While both behavioral therapy and medication demonstrated similar rates of improvement, it is worth noting that compliance rates were found to be low [10]. A Cochrane review examined the data on the benefits of employing device-mediated biofeedback in combination with PFMT versus PFMT alone. While the results showed that biofeedback may be more beneficial than PFMT alone, it is crucial to remember that numerous comparisons were impacted by other variables, making the conclusions less definitive [10]. Other potential explanations for the differences identified among biofeedback participants were longer treatment durations, higher therapist engagement, and modifications in PFMT protocols. Biofeedback's efficacy with PFMT was unclear [10]. The Optimal PFMT for Adherence Long-Term (OPAL) study examined the efficacy of PFMT with electromyographic biofeedback in clinical and home settings. The goal was to evaluate whether this combination would reduce incontinence severity in females with stress or mixed UI better than PFMT alone [10].

## Review

Methodology

PubMed, Google Scholar, and the Cochrane Library were used to collect database using the following terms: ("Urinary Incontinence/prevention and control"[Majr:NoExp] OR "Urinary Incontinence/psychology"[Majr:NoExp] OR "Urinary Incontinence/rehabilitation"[Majr:NoExp] OR "Urinary Incontinence/surgery"[Majr:NoExp] OR "Urinary Incontinence/therapy"[Majr:NoExp] OR "Urinary Incontinence/urine"[Majr:NoExp]) AND "Pelvic Floor/physiology"[Majr:NoExp] AND ("Urinary Incontinence, Stress/prevention and control"[Majr:NoExp] OR "Urinary Incontinence, Stress/rehabilitation"[Majr:NoExp] OR "Urinary Incontinence, Stress/surgery"[Majr:NoExp] OR "Urinary Incontinence, Stress/therapy"[Majr:NoExp] OR "Urinary Incontinence, Stress/urine"[Majr:NoExp]).

Study Selection

Randomized clinical trials (RCTs) that satisfied the effectiveness of creatine (Cr) in enhancing metabolic performance were considered. To assess eligibility, two investigators carefully read the full title and content of each RCT. We selected the latest literature and articles published in the past 10 years, including papers written in the English language, or if the full free-text English-language translation is available. RCTs were excluded if the full text of the papers could not be retrieved. RCTs focusing on the outcomes obtained from Cr as a supplement were strictly chosen. Gray literature and proposal papers were also not included. We used the Cochrane risk of bias assessment tools for RTCs.

Statistical Analysis

RevMan version 5.4 (2020) (The Cochrane Collaboration, The Nordic Cochrane Centre, Copenhagen, Denmark) was utilized for all statistical analyses. The mean difference with 95% confidence intervals (CIs) was used to present the trial results, and an odds ratio effects model was used to pool them. The method outlined by Mantel-Haenszel et al. was used to calculate the standard deviations (SDs) or standard errors if they were not reported in the trial. In light of the possible high between-study variance due to different study designs and populations, we used a fixed effects model rather than a random effects model.

Forest plots were generated to evaluate the pooling results visually. Any differences between the subgroups were found using the chi-square test. Higgins I2 was used to measure study heterogeneity, and a value of less than 50% was considered acceptable. To assess publication bias, a visual inspection of the funnel plot was utilized. A significance level of less than 0.05 was considered to account for each case.

Results

Search Results

**Figure 1:**
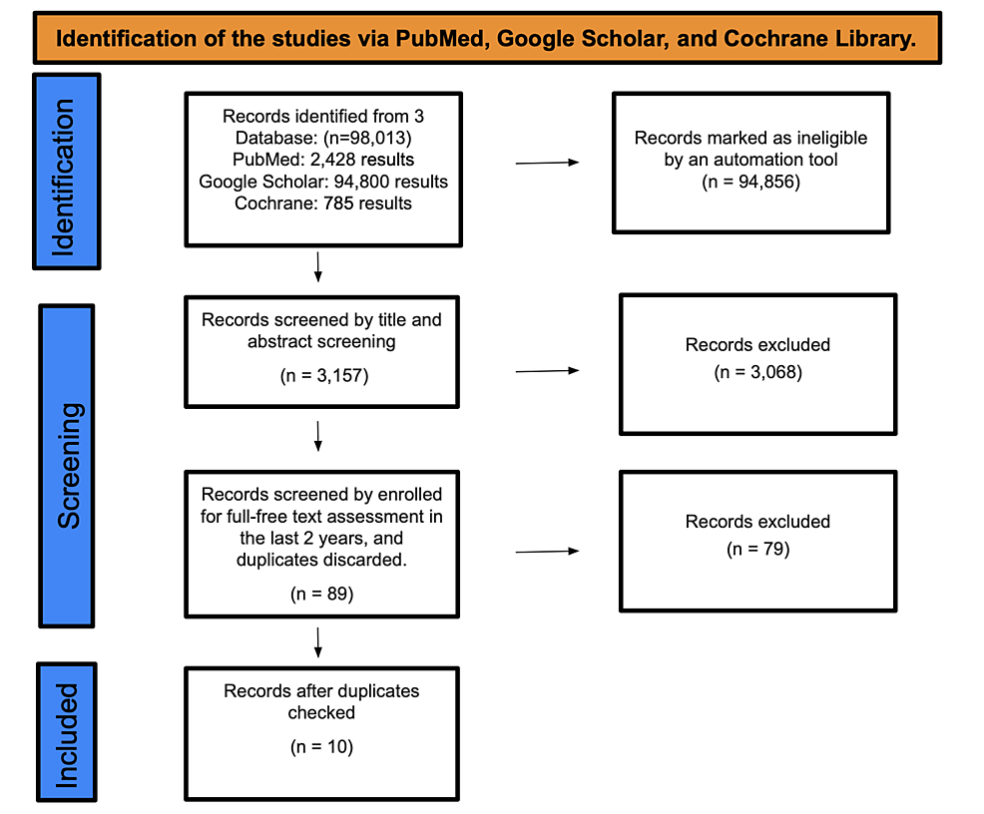
Identification of studies via databases and registers

A total of 98,013 studies were found after searching PubMed, Google Scholar, and the Cochrane Library. A total of 94,856 studies were marked as ineligible by an automation tool. There were a total of 3,157 studies that underwent title and abstract screening, with 3,068 papers being discarded. The remaining 89 papers were chosen by full-free text evaluation in the previous five years, and after discarding duplicates, resulting in the elimination of 79 studies, only 10 studies were enlisted for the final collection of data (Figure 1).

Table 1 presents an in-depth description of the articles we decided to use.

**Table 1:** Data extraction RCT: randomized clinical trial, KT: Kegel Training, RP: radical prostatectomy, ICIQ-UI SF: International Consultation on Incontinence Questionnaire-Urinary Incontinence Short Form, PFMT: pelvic floor muscle training, SUI: stress urinary incontinence, MUI: mixed urinary incontinence, QOL: quality of women’s lives, BFB: biofeedback, PPI: postoperative pain intensity

Author	Year of publication	Study design	Quality tool	Primary research	Outcome evaluation
Johannessen et al. [11]	2021	RCT	Cochrane risk of bias assessment tool	This study enrolled over 18 pregnant females with one viable fetus. Females in the exercise group performed a 12-week standardized pelvic floor muscle training program.	Our data suggests that pregnant females should exercise. We also found PFMT effective in prenatal exercise.
Hagen et al. [12]	2020	RCT	Cochrane risk of bias assessment tool	From February 2014 to July 2016, 600 females over 18 were examined for stress or mixed urine incontinence.	Biofeedback PFMT had an average ICIQ-UI SF score of 8.2 after 24 months, whereas PFMT had an 8.5.
Hagen et al. [13]	2020	RCT	Cochrane risk of bias assessment tool	Females > 18 with new mixed urinary incontinence or stress incontinence were included.	A study-related incident occurred in 23 of 48 participants, while the rest suffered non-serious adverse events.
Dumoulin et al. [14]	2020	RCT	Cochrane risk of bias assessment tool	Eight females participated in a 12-week PFMT program.	Elderly females with stress and mixed urine incontinence benefited from individual and group PFMT.
Zachovajeviene et al. [15]	2019	RCT	Cochrane risk of bias assessment tool	Between September 2010 and May 2012, 148 Caucasian males were randomly assigned to treatment groups.	Exercise of the diaphragm, abdominal wall, and pelvic floor muscles affected strength and endurance differently. They all reduced radical prostatectomy-induced urine loss in males.
Ptak et al. [16]	2019	RCT	Cochrane risk of bias assessment tool	SUI affected 137 females in the study. The study aims to assess the PFMT QOL questionnaire's efficacy.	Females with stress urinary syndrome may improve their quality of life by training the PFM, synergistic muscles, and individual PFM workouts.
Milios et al. [17]	2019	RCT	Cochrane risk of bias assessment tool	A control group of 47 RP patients received low-volume treatment, whereas an intervention group of 50 received intervention.	Prior to prostate surgery, pelvic floor exercises began. The study found that it improved pelvic floor muscle function, reduced PPI, and improved incontinence QOL.
de Lira et al. [18]	2019	RCT	Cochrane risk of bias assessment tool	Our institution accepted all prostate cancer patients aged 45-75 for RP from March 2013 to December 2014.	A preliminary review three months after open retropubic RP showed that two supervised PFMT BFB sessions before surgery did not improve continence or erectile function.
Jha et al. [19]	2018	RCT	Cochrane risk of bias assessment tool	Women's urinary incontinence and sexual dysfunction were examined. The research compared pelvic floor muscle training to electric stimulation.	Physiotherapy improves sexual function in females with urine incontinence and sexual dysfunction.
Aydın Sayılan et al. [20]	2018	RCT	Cochrane risk of bias assessment tool	The procedure group conducted pelvic floor muscle exercises three times a day for six months. The control group did not exercise.	KT and pelvic floor muscle training had equal total continence rates three and six months post-RP.

Meta-analysis of outcomes

**Figure 2:**
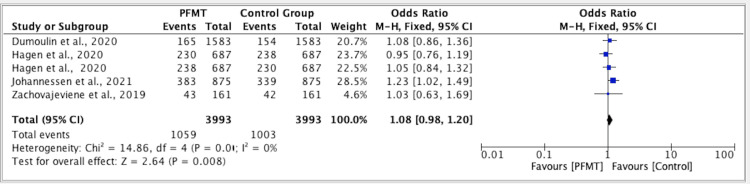
Forest plot for studies on the efficacy of PFMT versus the control group PFMT: pelvic floor muscle training, CI: confidence interval References: [11-15]

The results of five studies showed an odd ratio of 1.08 for the efficacy of PFMT versus the control group. The odd ratio was 1.08 (fixed effect, 95%). The CI was 0.98-1.20, the P value was 0.01, and the heterogeneity (I2) was 0% (Figure 2).

**Figure 3:**
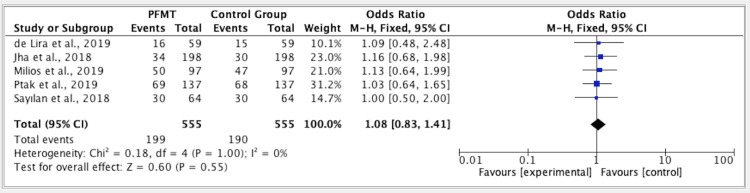
Forest plot for studies on the efficacy of PFMT versus the control group PFMT: pelvic floor muscle training, CI: confidence interval References: [16-20]

The results of five studies showed an odd ratio of 1.08 in the efficacy of PFMT versus the control group. The odds ratio was 1.08 (fixed effect, 95%). The CI was 0.83-1.41, the P value was 1.00, and the heterogeneity (I2) was 0% (Figure 3).

**Figure 4:**
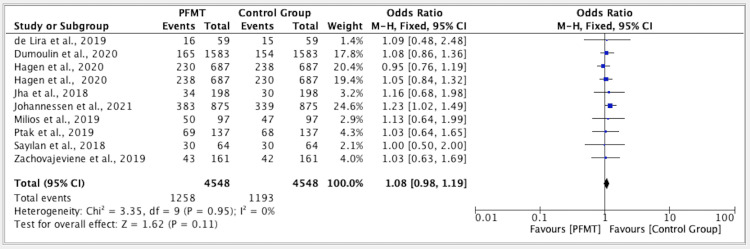
Forest plot for studies on the overall efficacy of PFMT versus the control group PFMT: pelvic floor muscle training, CI: confidence interval References: [11-20]

The results of five studies showed an odd ratio of 1.08 in the overall efficacy of PFMT versus the control group. The odds ratio was 1.08 (fixed effect, 95%). The CI was 0.98-1.19, the P value was 0.95, and the heterogeneity (I2) was 0% (Figure 4).

**Figure 5:**
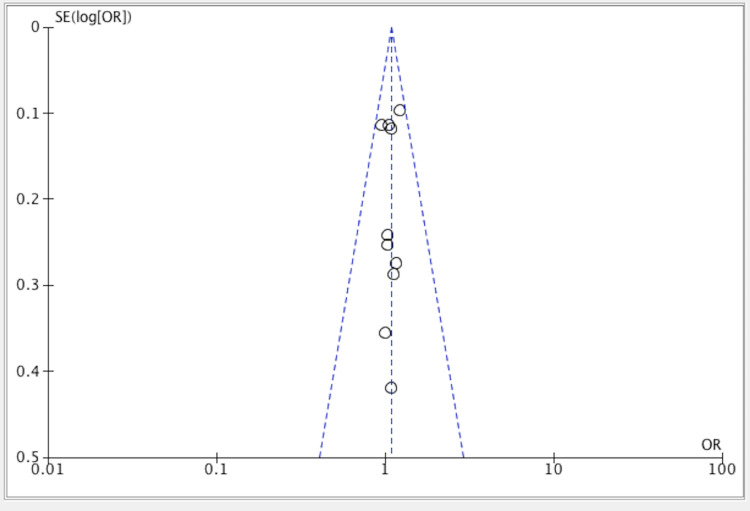
Forest plot for all included studies on the efficacy of PFMT versus the control group PFMT: pelvic floor muscle training References: [11-20]

Figure 5 shows publication bias representation through the funnel plot method.

Discussion

Johannessen et al. [11] performed an RCT to show the advantages of participation in a prenatal exercise program that included PFMT to reduce the risk of UI three months after delivery. The most significant effect of PFMT was seen in females who had incontinence at the start of the research. Additional research discovered that certain characteristics were connected to an increased incidence of UI three months after giving birth. Age, having UI during late pregnancy, having a birth weight of 4,000 g or greater, and having obstetric anal sphincter injuries are all risk factors. A cesarean delivery, on the other hand, was observed to dramatically reduce the incidence of UI three months postpartum [11]. The investigation of the continued influence of a prenatal exercise program that incorporates PFMT three months after delivery is a unique component of this study [11]. In prior research, a 12-week exercise program that incorporated PFMT showed a favorable influence on the prevalence of UI in late pregnancy. The research found that 42% of females in the intervention group had some degree of UI during pregnancy weeks 32-36, whereas 53% of females in the control group did not (P = 0.004) [11]. Another RCT, according to Hagen et al. [12], the OPAL trial, demonstrates that biofeedback PFMT has a higher cost than basic PFMT, but it also results in a larger number of quality-adjusted life years. It should be emphasized, however, that none of these findings were statistically significant [12]. The female participants were enthusiastic about both treatments. Both therapies received equivalent levels of commitment, and the desire to address their UI symptoms aided their involvement. However, one consistent barrier that the females encountered was a lack of time to fully participate in the treatments [12].

Another study undertaken by Hagen et al. [13] found no statistically or clinically significant difference in the severity of PFMT. The groups did not differ significantly in several secondary urine outcomes at the 24-month follow-up. The outcomes included the cure or improvement of lower urinary tract symptoms, the influence on condition-specific quality of life, and the patient's perspective on UI improvement. Other critical secondary outcomes, such as pelvic floor muscle function, prolapse symptoms, and the need for further treatment for UI, did not vary significantly across groups [13]. In an RCT conducted by Dumoulin et al. [14], of 362 females, 319 successfully completed the one-year follow-up. The research discovered that females who received individual PFMT saw a 70% decrease in UI episodes, while those who received group-based PFMT experienced a 74% reduction. Individual PFMT and group-based PFMT were shown to be equally efficacious for all secondary outcomes after one year [14]. The research by Zachovajeviene et al. [15] offered a unique strategy for activating PFMT that improved pelvic floor muscle strength (PFMS), pelvic floor muscular endurance (PFME), and UI in males after RP. Males who engage in diaphragm muscle training, abdominal muscle training, and PFMT programs after RP have persistent and significant improvements in PFMS and PFME. Patients following RP may benefit from one of the three training regimens indicated for urinary incontinence rehabilitation [15].

In their analysis, Ptak et al. [16] showed that conservative treatment using the A training program (PFMT + transversus abdominis (TrA) muscle) led to much better quality-of-life results than the B program (PFMT). These gains were shown in areas such as the capacity to execute household duties and participate in physical activities outside the home, as well as the ability to travel and interact. Other areas that showed improvement included control over fluid intake, feelings of embarrassment, fatigue, anxiety related to unpleasant odors, sleep problems, frequency of changing panty liners, changing wet underwear, and emotions [16]. Milios et al. [17] discovered in their RCT that patients who received more rigorous PFMT utilizing standing postures had good results. When compared to a control group that used a different technique, these results included less leakage, quicker restoration to continence, and a higher quality of life [17]. The findings of this research revealed that a higher-intensity prehabilitation session had good benefits. However, we discovered that the control group recovered naturally following the procedure. The active rehabilitation technique that focused on strengthening the physiological function of both fast- and slow-twitch muscle fibers improved recovery time [17]. According to the findings of de Lira et al. [18], the results of their RCT did not show any significant positive effects during an initial evaluation conducted after three months. This assessment depends on the patient's replies to particular UI and erectile dysfunction questionnaires. So, without the direct supervision and input of a physical therapist, we don't know how well a three-month PFMT program improves pelvic muscle strength, helps muscles recover from possible damage after RP, and helps restore urinary continence [18].

Jha et al. [19] performed a trial in which 114 females were randomly allocated to either the intervention or control groups. At the follow-up, the researchers calculated the mean scores for prolapse and incontinence sexual function questionnaire dimensions. The intervention group scored 33.1 (SD: 5.5) for prolapse and 32.3 (SD: 5.2) for incontinence, whereas the control group scored higher, suggesting greater sexual function [19]. To conclude, no differences in secondary outcomes were identified across groups throughout the follow-up period. Physiotherapy, on the other hand, has been shown to improve total sexual function in females who have UI as well as sexual dysfunction [19]. In a recent study conducted by Aydın Sayılan et al. [20], it was found that the total incontinence consultation on the incontinence-short form scores, which are used to objectively assess individuals with incontinence issues, decreased as time progressed. The drop in the third and sixth months was determined to be statistically significant. Patients who have undergone RP may find pelvic muscle floor exercises helpful in managing postoperative incontinence [20].

## Conclusions

In this meta-analysis, we analyzed the effectiveness of PMFT in different clinical scenarios. From them, we have postpartum, post-radical prostatectomy, or clinical UI alone. We establish that PFMT has a protective effect on UI at three months postpartum. This depends on various risk factors, such as if the patient underwent a cesarean section or a normal delivery. Some of the study outcomes were based on group PFMT versus individual training, in which the last one was more effective than the group PFMT. PFMT alone was not as good as the PFMT + TrA training, which was directly related to an enhancement of muscle strength and endurance that had good outcomes in their daily activities. However, from the studies recollected, there were three that had no statistical significance during the RCTs when comparing PFMT versus the control group.
